# A retrospective analysis on the transmission of *Plasmodium falciparum* and *Plasmodium vivax*: the case of Adama City, East Shoa Zone, Oromia, Ethiopia

**DOI:** 10.1186/s12936-019-2827-6

**Published:** 2019-06-11

**Authors:** Temesgen File, Hunduma Dinka, Lemu Golassa

**Affiliations:** 1 Department of Applied Biology, Adama Science and Technology University, P.O.Box 1888, Adama, Ethiopia; 2 Aklilu Lemma Institute of Pathobiology, Addis Ababa University, P.O.Box 1176, Addis Ababa, Ethiopia

**Keywords:** Adama City, *Plasmodium falciparum*, *Plasmodium vivax*, Retrospective, Urban malaria

## Abstract

**Background:**

Malaria is more often considered a problem of the rural poor and the disease has been overlooked in urban settings for centuries due to the assumption that economic development in urban areas results in better life conditions, such as improved housing, drainage system and environmental changes that makes urban areas not conducive for breeding of the malaria vector. But, for many African countries, including Ethiopia, in most urban areas, although there are rapid developments, they are characterized by poor housing, lack of sanitation and drainage of surface water that would provide favourable conditions for vector breeding. Limited studies have been conducted as far as urban malaria is concerned in Ethiopia. The purpose of this study was to assess the status of falciparum and vivax malaria transmission in Adama City, Eastern Shoa Zone, Oromia, Ethiopia. Understanding the local epidemiology of malaria will help policy makers and other stakeholders to design and implement tailored cost effective and efficient intervention strategies targeting urban malaria.

**Methods:**

The study was designed to analyse 5-year trends of malaria burden by two co-endemic species in Ethiopia (*Plasmodium falciparum* and *Plasmodium vivax*) and its annual and seasonal transmission pattern in the city, by using retrospective data on malaria burden by species, malaria related inpatient department (IPD) and outpatient department (OPD) consultations from 2013/14 to 2017/18.

**Results:**

OPD retrospective data analysis indicated that adolescents and adults (≥ 15 years of age) were most affected by *P. vivax* 43.5% (2986/6862) and *P. falciparum* 31.7% (2179/6862). *Plasmodium vivax* was found to be a predominant species in causing malaria burden in the city exhibiting less seasonal occurrence, and the relative burden of *P. vivax* is gradually increasing from year to year over *P. falciparum.*

**Conclusion:**

Malaria is endemic to the city showing a public health problem. The productive group of the community, adolescents and adults, were most affected exacerbating poverty. *Plasmodium vivax* was found to be the highest malaria burden in the city and the observed epidemiological shift from *P. falciparum* to *P. vivax* calls for additional tailored intervention strategies to reduce the associated burden.

## Background

Malaria is a parasitic disease that shows considerable spatial heterogeneity globally [1, 2], regionally [3–5] and locally. According to WHO, the African region accounts for 90% of global malaria cases and 90% of malaria deaths [6, 7]. In 2016, sub-Saharan Africa (SSA) accounted for 216 million malaria cases and 445,000 malaria deaths mostly in young children [8]. It has been reported that in Africa alone the cost of illness, treatment and premature death due to malaria of at least USD$12 billion per year contributes significantly to poverty [6, 9]. Of the four most common species of Plasmodia that infect humans, the vast majority of deaths in SSA are caused by *Plasmodium falciparum*, while *Plasmodium vivax*, *Plasmodium ovale* and *Plasmodium malariae* cause generally milder forms of malaria [10–13]. *Plasmodium vivax* is responsible for most malaria cases in Asia and Latin America, but is almost absent from most of Central Africa due to absence of Duffy antigen receptor that the parasite uses to invade human erythrocytes. However, this interpretation is challenged by recent findings of *P. vivax* infection in Duffy-negative people in different part of Africa [14].

Ethiopia is unique from many African countries in that *P. vivax* is co-endemic with *P. falciparum* at approximately equal case incidence rate [15, 16]. It has been reported that the major malaria transmission season in Ethiopia is from September to December, following the main rainy season from June to September. The shorter transmission season spans April to May following the shorter rainy season in some parts of the country [17].

Although malaria is typically considered mainly a problem of the rural poor, this disease has been a concern in urban settings for centuries [18]. Evidence suggests that economic development, improved housing, drainage system of *Anopheles* breeding sites, household mosquito proofing, expanded personal protection, effective diagnosis and treatment, and other factors that exist in urban areas have contributed to the recent global decline in malaria incidence [18, 19]. Africa’s demography is rapidly changing, with an increasing number of people moving to urban areas [20], with poor infrastructure and economic development. Like other developing countries, Ethiopian urban centers are characterized by a poorly developed economic base, high level of unemployment and incidence of poverty and slum habitation [21, 22]. Consequently, malaria transmission persists and in some cases at even higher levels than in surrounding rural areas [20]. A recent analysis estimated that urban SSA may account for 6–28% of the global malaria burden [23]. As urbanization increases, factors that contribute to urban malaria become more important. The aim of this study was to assess the status of falciparum and vivax malaria transmission and its risk factors in Adama town. Understanding such local epidemiology of malaria will help policy makers and other stakeholders to design and implement a cost-effective and efficient intervention strategy of malaria.

## Methods

### Description of the study area

**Fig. 1:**
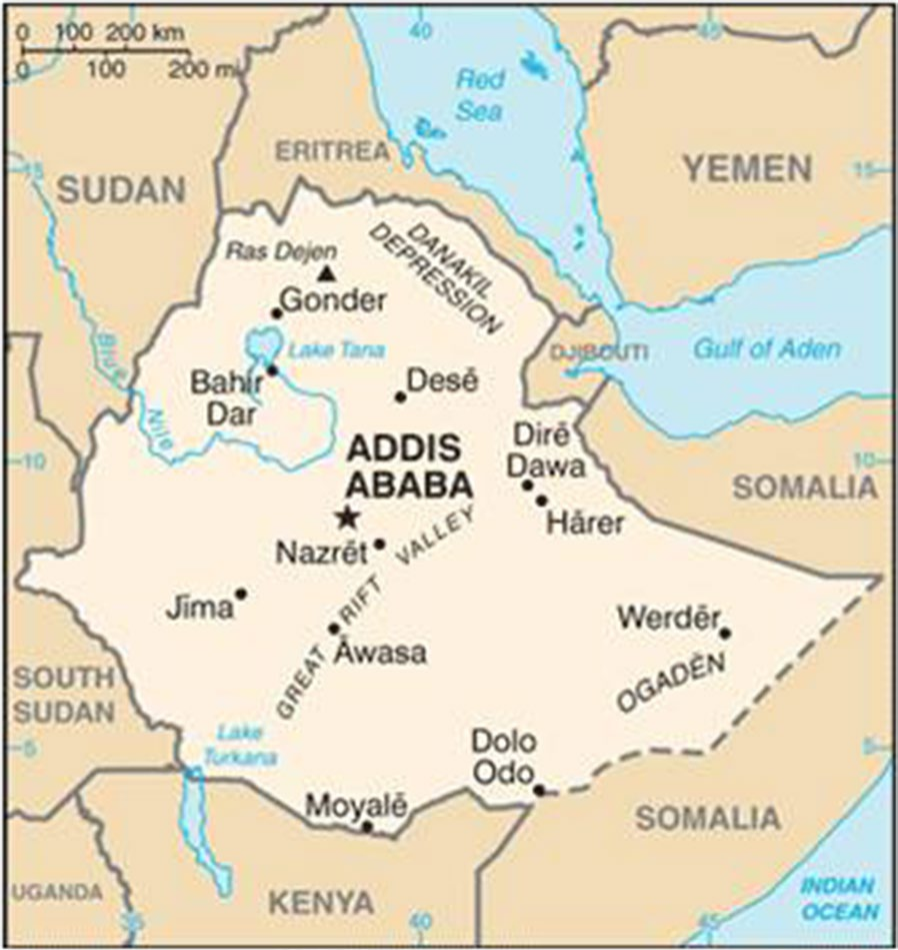
Physical location of Adama/Nazareth town

Adama town is located at 8.54°N and 39.27°E, at elevation of 1712 m, about 99 km southeast of Addis Ababa. The city is located between the base of an escarpment to the west, and the Great Rift Valley to the east [24] (Fig. 1). It is a rapidly growing major city near Addis Ababa in central Ethiopia, with a total population of 500,000 in 2018. Due to its elevation < 2000 m above sea level and various factors that favour mosquito breeding, Adama is a well-known malarious town in Ethiopia. It is surrounded by hills in the north, east and west, and commonly affected by flooding during the rainy season. The rainfall pattern is heavy from mid-June to mid-September with a shorter rainy season in March. The seasonal temperatures are 16–32 °C, and favourable for the breeding of *Anopheles arabiensis* (the predominant malaria vector in the region) [25]. Plenty of bushy gorges, ditches, shanty and slum dwellings, untidiness, and vegetation-covered residential areas are some of the common epidemiological factors that favour mosquito breeding in the city.

The health facilities in Adama include: government-owned teaching hospital, private hospitals, government-owned health centres, non-government organization-based health centre run by St Francisco Catholic Mission, one government-owned malaria diagnostic centre and private or specific organization-owned clinics having different status (higher, medium, primary) providing limited services to their customers.

### Study design and data collection

Retrospective laboratory record data of malaria cases were obtained from the database of Adama City administration health offices. The source of the data was microscopically confirmed malaria cases screened by qualified medical laboratory technologists. The database consists of 5-year malaria inpatient department (IPD) and outpatient department (OPD), and the total malaria cases by facility, which are regularly updated and reported to the central surveillance system. The data were accessed after approval by the ethical clearance protocol of this study from Adama Science and Technology University (ASTU).

### Data analysis

Microscopically confirmed malaria cases were systematically collected from database of Adama City health bureau. Data were checked for completeness and consistency, and double-entered into Microsoft Excel 2016 version. Descriptive data such as bar graph and line graphs were used to show the seasonal and yearly trends of malaria epidemiology.

## Results

**Fig. 2:**
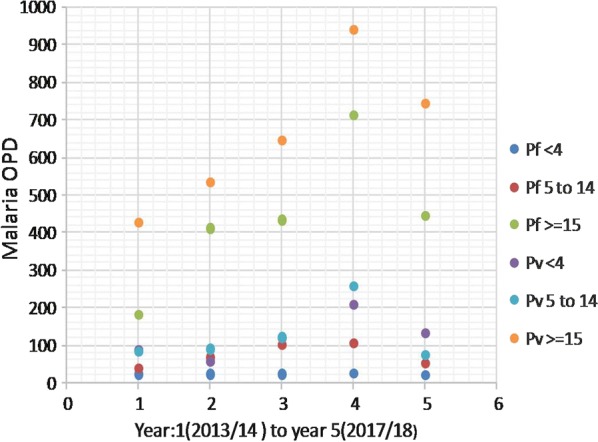
Five-year retrospective OPD data on two co-endemic *Plasmodium* species in Adama City (Ethiopia) from 2013/14 to 2017/18

**Fig. 3:**
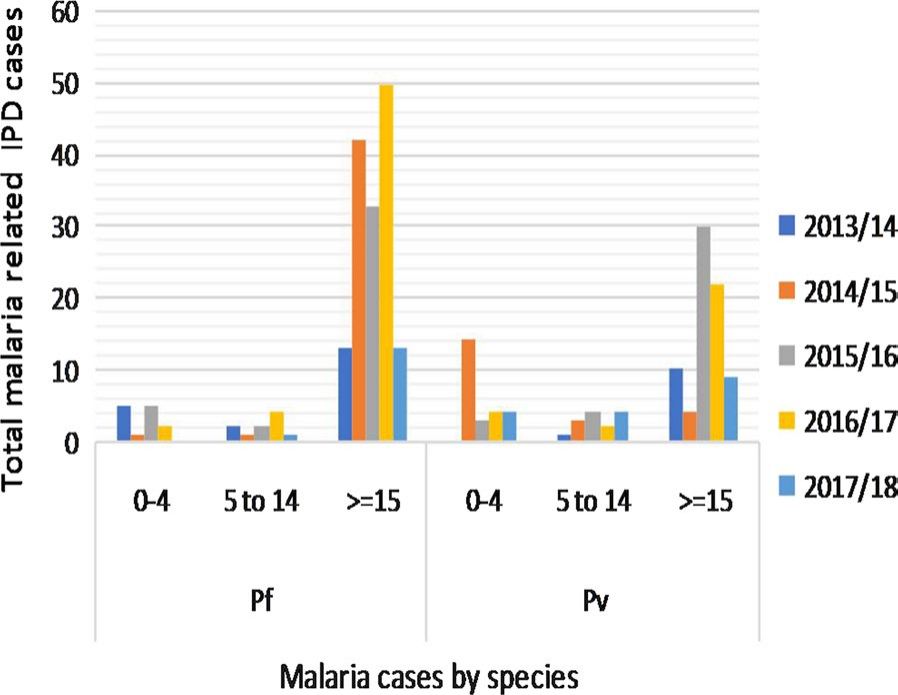
Five-year IPD data on the trends of malaria cases in two co-endemic *Plasmodium* species in Adama City (Ethiopia) from 2013/14 to 2017/18

OPD retrospective data (Fig. 2) indicated that a total of 6862 malaria cases were reported from 35 health facilities (21 clinics, 9 health centres, 4 hospitals, and 1 malaria diagnostic centre) to the city health bureau. Adolescent and adult (≥ 15 years) age groups were the most affected by both *P. vivax*, and *P. falciparum*, followed by school-aged early adolescents (5–14 years old). Contrary to the national report, the relative burden of *P. vivax* is gradually increasing from year to year over *P. falciparum* (Fig. 2) compared with similar age group. Similarly, IPD data of admitted patients to the health facilities due to malaria case during the same year depicts adolescent and adults (≥ 15 years) were the most affected followed by pre-school children (0–4 years) (Fig. 3). The highest malaria related morbidity is accounted for the severe malaria (*P. falciparum*) over that of *P. vivax.*

**Fig. 4:**
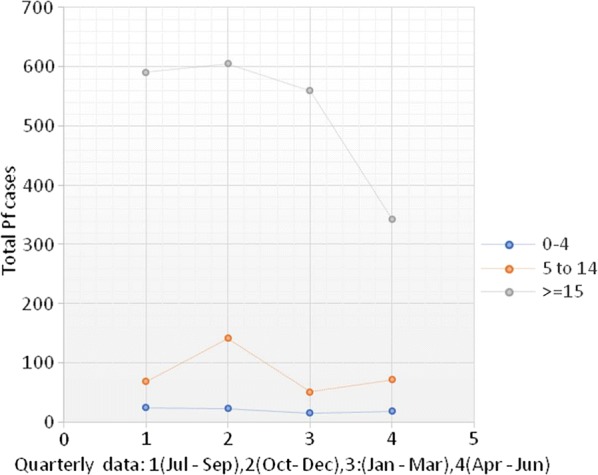
Average seasonal trends of *Plasmodium falciparum* infection by age from 2013/14 to 2017/18 in Adama City

**Fig. 5:**
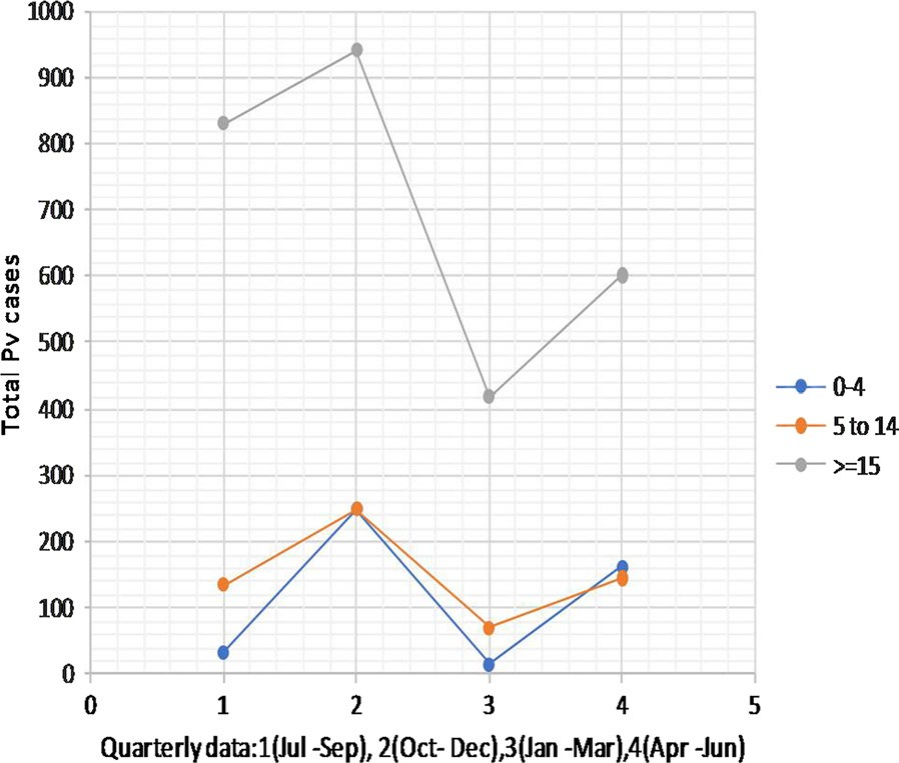
Average monthly trends of *Plasmodium vivax* infection by age from 2013/14 to 2017/18 in Adama City

During the study period, it was observed that annual trends for the disease pattern for co-endemicity of *P. falciparum* and *P. vivax* cases (Figs. 4 and 5) revealed malaria is endemic to the city. The highest number of cases was noted from October to December whereas the fewest were around March and April for both *P. falciparum* and *P. vivax*.

## Discussion

Despite the fact that numerous studies have been conducted in Ethiopia at national and regional level in expanding malaria interventions, malaria is still a leading communicable disease, causing morbidity and mortality. *Plasmodium falciparum* and *P. vivax* co-exists, accounting for 60 and 40% of all malaria cases, respectively [15]. Contrary to the national report, in Adama City out of 6862 malaria cases reported from OPD data from 2013/14 to 2017/18 in retrospective study, 61% was *P. vivax* and 39% was *P. falciparum.* In the present study, as malaria control and prevention efforts have been intensified, the epidemiological shift of malaria burden from *P. falciparum* to *P. vivax* was observed from 2013/14 to 2017/18 in the city. This finding is consistent with the report of Ethiopian Federal Ministry of Health, which states that *P. vivax* was the main causative agent of malaria in Oromia regional state of Ethiopia [18]. This again strongly supports the previous study done in Adama in 2015 by Golassa and White [25], where they concluded 70% of *P. vivax* infections were suggested to have arisen from relapse during February to April, and 40% from August to October 2015. The current rise in the number of cases of *P. vivax* in the city might be due to misdiagnosis of the parasite in the past or the emerging trend of the parasite clone to infect Duffy-negative individuals [14] and relapse cases. The issue needs further investigation.

Malaria is a disease of poverty, and contributes to national poverty through its impact on foreign direct investment, tourism, labour productivity and trade [26]. Household level impact includes income loss due to cost of treatment, absence from work by patient and caregiver, and funeral expenses due to malaria-related mortality, and greatly affects the productive age of the community exacerbating poverty [26]. The present study revealed that the productive group of the society (≥ 15 years of age) is most affected, compared to other age groups. This finding differs from the report of Golassa and White [27] where they concluded that 6–15 years of age were the most affected (49.5%) group, and pre-school children below 5 years of age were the most affected by *P. vivax.* However, their study was on a population level estimate of *P. vivax* blood stage while their study site was limited to Adama malaria diagnostic centre, which might have contributed to such differences.

Understanding spatial and temporal variation in vector density and transmission intensity is useful in planning effective malaria control programmes and determining the optimum allocation of limited resources [8, 27]. It has been reported that the major malaria transmission season in Ethiopia is from September to December following the main rainy season from June to September. The shorter transmission season spans from April to May following the shorter rainy season in some part of the country [17]. This is near agreement with the present study where unstable temporal variation was observed with higher transmission from October to December, and minimum transmission between March and April.

## Conclusion

Malaria is endemic to the city although there is a possibility of limited transmission rural to urban or vice versa causing a public health problem. The productive groups of the community, adolescents and adults above 15 years of age, are most affected by malaria. Malaria negatively affects productivity and exacerbates poverty due to treatment cost, income loss by patients and caregivers. More malaria cases were recorded between October and December and fewer cases were noted between March and April. *Plasmodium vivax* contributed to the highest malaria burden in the city, which might be due to the possibility of relapse and emerging trends of *P. vivax* in Duffy-negative individuals. Since the data for this study are mainly by microscopic method, molecular evidence is highly recommended for accurate species identification and parasite genotyping, which can provide useful data in complementing traditional epidemiological surveys to design effective malaria control strategy at local and national level.

## Data Availability

The authors confirm that all data underlying the findings are fully available without restriction. All relevant data are within the manuscript.

## Abbreviations

IPD: in-patient department
OPD: out-patient department
SSA: sub-Saharan Africa
WHO: World Health Organization
ASTU: Adama Science and Technology University
